# Clinical research challenges posed by difficult-to-treat depression

**DOI:** 10.1017/S0033291721004943

**Published:** 2022-02

**Authors:** A. John Rush, Harold A. Sackeim, Charles R. Conway, Mark T. Bunker, Steven D. Hollon, Koen Demyttenaere, Allan H. Young, Scott T. Aaronson, Maxine Dibué, Michael E. Thase, R. Hamish McAllister-Williams

**Affiliations:** 1 Duke-NUS Medical School, Singapore; 2 Department of Psychiatry and Behavioral Sciences, Duke University, Durham, NC, USA; 3 Department of Psychiatry, Texas Tech University, Permian Basin, TX, USA; 4 Departments of Psychiatry and Radiology, Columbia University, New York, NY, USA; 5 Department of Psychiatry, Washington University in St. Louis, St. Louis, MO, USA; 6 LivaNova USA PLC, Houston, TX, USA; 7 Departments of Psychology and Psychiatry, Vanderbilt University, Nashville, TN, USA; 8 University Psychiatric Center, KU Leuven, Leuven, Belgium; 9 Faculty of Medicine, KU Leuven, Leuven, Belgium; 10 Department of Psychological Medicine, Institute of Psychiatry, Psychology and Neuroscience, King's College, London, UK; 11 Department of Clinical Research, Sheppard Pratt Health System, Baltimore, MD, USA; 12 Department of Neurosurgery, Heinrich Heine University Düsseldorf, Düsseldorf, Germany; 13 Medical Affairs Europe, LivaNova Deutschland GmbH, Munich, Germany; 14 Department of Psychiatry, University of Pennsylvania, Philadelphia, PA, USA; 15 Northern Centre for Mood Disorders, Newcastle University, Newcastle upon Tyne, UK; 16 Cumbria, Northumberland, Tyne and Wear NHS Foundation Trust, Newcastle upon Tyne, UK

**Keywords:** Classification, difficult-to-treat depression, outcome measures, study design, taxonomy, treatment-resistant depression

## Abstract

Approximately one-third of individuals in a major depressive episode will not achieve sustained remission despite multiple, well-delivered treatments. These patients experience prolonged suffering and disproportionately utilize mental and general health care resources. The recently proposed clinical heuristic of ‘difficult-to-treat depression’ (DTD) aims to broaden our understanding and focus attention on the identification, clinical management, treatment selection, and outcomes of such individuals. Clinical trial methodologies developed to detect short-term therapeutic effects in treatment-responsive populations may not be appropriate in DTD. This report reviews three essential challenges for clinical intervention research in DTD: (1) how to define and subtype this heterogeneous group of patients; (2) how, when, and by what methods to select, acquire, compile, and interpret clinically meaningful outcome metrics; and (3) how to choose among alternative clinical trial design options to promote causal inference and generalizability. The boundaries of DTD are uncertain, and an evidence-based taxonomy and reliable assessment tools are preconditions for clinical research and subtyping. Traditional outcome metrics in treatment-responsive depression may not apply to DTD, as they largely reflect the only short-term symptomatic change and do not incorporate durability of benefit, side effect burden, or sustained impact on quality of life or daily function. The trial methodology will also require modification as trials will likely be of longer duration to examine the sustained impact, raising complex issues regarding control group selection, blinding and its integrity, and concomitant treatments.

## Introduction

Over one-third of persons with major depressive disorder (MDD) will not achieve sustained symptom remission after several treatment trials (Agency for Healthcare Research and Policy (AHQR), 2011). The Sequenced Treatment Alternatives to Relieve Depression (STAR*D) trial found that only two-thirds of patients reached remission after four treatment steps. Furthermore, the relapse rate over the year subsequent to remission ranged from 35% to 70%, increasing with the number of acute treatment trials needed to achieve remission (Rush et al., 2006b).

These and other findings formed the basis for the heuristic, treatment-resistant depression (TRD), which is typically defined by the number of previously failed acute phase treatment trials, based on lack of short-term improvement in overall depressive symptom severity (Fava, 2003b; Sackeim, 2001; Thase & Rush, 1995). Various reports have used thresholds ranging from 1 to 4 or more failed acute phase treatment trials to define various levels of treatment resistance (Agency for Healthcare Research and Policy (AHQR), 2011; Conway, George, & Sackeim, 2017; Lisanby et al., 2009). While the FDA recognizes the utility of TRD in its approval and labeling of interventions, the heuristic poses a myriad of clinical and research challenges (McAllister-Williams et al., 2020; Rush, Aaronson, & Demyttenaere, 2019).

Favoring the concept of TRD is the fact that the degree of resistance seems to be easily assessed (Berlim & Turecki, 2007b; Sackeim et al., 2019). Generally, the likelihood of response or remission with subsequent treatments decreases with increasing numbers of previously failed acute phase treatment trials (Heijnen, Birkenhager, Wierdsma, & van den Broek, 2010; Lisanby et al., 2009; Prudic et al., 1996; Rush et al., 2006b), while the likelihood of relapse increases if short-term remission is obtained (Prudic et al., 2013; Rasmussen et al., 2009; Rush et al., 2006b; Sackeim et al., 1990).

On the other hand, closer examination of the concept of TRD presents challenges. For example, what defines a failed trial: lack or response or lack of remission? What constitutes an adequate trial? What about patients who markedly improve but do not stay better? Or those who cannot tolerate a medication (is that a failed trial)? Must all the failed trials occur in the current episode or do failed treatments in prior episodes also count, especially since a treatment that failed in the past is expected to fail again if tried in a new episode? All of these questions can be operationalized and attempts have been made to do this on the basis of a Delphi consensus approach (Sforzini, 2021). Nevertheless, there is inevitably great diversity in the prior ‘failed’ treatments in individuals with TRD, such that there is no expectation of biological or etiological homogeneity in any group defined solely by TRD. Indeed, definitions of TRD generally don't include non-pharmacological treatments and rarely, if ever, include psychotherapy or psychosocial interventions. Consequently, the treatment implications of TRD are largely nonspecific (i.e. more prior failed trials reduce hopefulness about future therapeutics). Finally, TRD has no practical, actionable clinical implications other than to suggest attempting another, primarily pharmacological, treatment trial with a different intervention or combination.

In recognition of these limitations, a new clinical heuristic, difficult-to-treat depressions (DTDs), has been proposed to stimulate the timely identification and personalized management of patients for whom our current treatments – even if well-delivered and tolerated – are unlikely to either initiate or sustain symptomatic remission (McAllister-Williams et al., 2020; Rush et al., 2019). Patients with DTD often present with either chronic depressive symptoms that are insufficiently relieved by treatment changes, or with symptoms that seemingly improve, at least temporarily, but the sustained benefit is not achieved. In either case, persons with DTD have substantially impaired daily function and poor quality of life (QoL) (Jaffe, Rive, & Denee, 2019; McAllister-Williams et al., 2020; Rush et al., 2019). They are also high utilizers of mental and general health services in both outpatient and inpatient settings (Kubitz, Mehra, Potluri, Garg, & Cossrow, 2013; Olchanski et al., 2013), resulting in high health costs that often persist for years (Amos et al., 2018; Benson, Szukis, Sheehan, Alphs, & Yuce, 2020; Greenberg, Corey-Lisle, Birnbaum, Marynchenko, & Claxton, 2004; Olfson, Amos, Benson, McRae, & Marcus, 2018; Sussman, O'Sullivan, Shah, Olfson, & Menzin, 2019; Wang et al., 2005). DTD is also associated with substantial morbidity and mortality (Amital et al., 2013; Huang et al., 2020). Clinical trial findings and care system database analyses (Eaton et al., 2008; Huang et al., 2020; Jaffe et al., 2019; Sussman et al., 2019) suggest that approximately 15–25% of depressed patients present with DTD, though its true prevalence and diagnostic boundaries remain uncertain.

A recent international consensus report detailed the clinical features and treatment implications of DTD, and suggested key principles for management (McAllister-Williams et al., 2020). When DTD is suspected, psychiatric, medical, and neuropsychological re-evaluations are recommended to identify potentially treatable causes of the depressive episode. If the depression is not improved following these efforts, DTD is ‘confirmed’ and the treatment goals might need to shift from the pursuit of symptomatic remission to optimizing symptom control, maximizing psychosocial function and QoL, and reducing the risk of deterioration and relapse (McAllister-Williams et al., 2020; Rush et al., 2019), while keeping an eye out for newly developing treatments that may be useful for the patient.

The concept of DTD acknowledges that our therapeutic armamentarium does not presently achieve sustained symptom remission in a significant proportion of depressed patients. Our limited armamentarium may be due, in part, to the heavy reliance on short-term (6–12 week) trials in ‘treatment-responsive’ populations when developing therapeutics for major depressive episodes (MDEs). These trials typically compare an antidepressant against a placebo, sham, or active comparator, and a statistically and clinically meaningful acute antidepressant effect is potentially identified. Subsequently, continuation or maintenance phase trials address prevention of relapse or recurrence (Frank et al., 1991; Rush et al., 2006a). However, these brief, symptom-focused approaches may be of limited relevance in DTD, as the pursuit of symptom remission through the administration of sequential monotherapies and treatment combinations delivered in a ‘try and try again’ approach typically results in diminishing returns and may seem futile to the clinician and patient (DeRubeis et al., 2020; Dunner et al., 2006; Hollon et al., 2014; Rush et al., 2006b).

At some point, clinical strategies useful in responsive populations are no longer optimal in DTD. This notion is not unique to DTD. In epilepsy, failure to benefit from two well-conducted trials of anticonvulsant medications is often viewed as the threshold for the diagnosis of medication-resistant epilepsy, triggering additional evaluations and potential surgical intervention (Jette, Reid, & Wiebe, 2014; Kwan & Brodie, 2010). These patients have only an estimated 3–5% chance of achieving at least 1 year of seizure remission with additional antiepileptic medication treatment, and seizure recurrence is common in those who achieve remission (Brodie, Barry, Bamagous, Norrie, & Kwan, 2012; Callaghan, Anand, Hesdorffer, Hauser, & French, 2007). Similarly, in STAR*D, after two failed treatment trials, the probability of achieving remission in MDD dropped 50% in the next two pharmacotherapy steps. Critically, similar to epilepsy, more failed acute-phase antidepressant medication trials result in both lower acute response/remission rates and higher rates of relapse during follow-up.

Thus, at some point, clinicians must decide whether to pursue DTD remission with another treatment trial or to change the aim of treatment to optimized symptom control, function, QoL, relapse mitigation, and treatment burden. This decision entails shared decision-making while considering each patient's aspirations, disease and treatment burdens (e.g. medical fragility), environmental circumstances, and anticipated risks and benefits of untried and sometimes minimally evaluated treatments, as well as other factors. This problem of when to change targets from sustained symptom remission to optimal patient and disease management is implicit in managing every depressed patient who is struggling despite multiple treatment attempts. The designation of DTD promotes a more deliberate, transparent and shared decision-making process. Further, it implicitly promotes the use of evidence-based interventions before embarking on less rigorously evidenced treatments.

There is a great need for clinical and service researchers to develop and evaluate patient-level clinical interventions for DTD, such as novel medications or combinations, psychotherapies, and neuro-stimulatory methods, as well as administrative/programmatic innovations such as intensive outpatient programs, peer-supported self-help programs aimed at promoting wellbeing, or substance use reduction programs.

To facilitate DTD intervention research, this report reviews three fundamental challenges: participant selection, outcome assessment, and study design. Participant selection is especially challenging because DTD is likely heterogeneous in etiology, course, pathobiology, and intervention responsiveness. The development of a DTD taxonomy could facilitate the identification of more homogeneous subgroups, thereby enhancing trial efficiency and, potentially, intervention targeting and staging (Sackeim, 2021). Outcome assessment concerns the selection of primary and secondary outcomes from multiple possibilities and how to efficiently collect, compile, and interpret these outcomes. Study design challenges include how to select among designs that optimize generalizability, while also preserving opportunities for making causal inference, selection of control conditions, study duration, and other issues.

## Challenges in identifying DTD patients for intervention trials

### What are the preferred evaluations when DTD is suspected?

The consensus report recommended that a broad set of evaluations be considered when DTD is suspected (McAllister-Williams et al., 2020) to identify overlooked modifiable causes of the depression. For example, depression may be a manifestation of undiagnosed endocrine disorders [e.g. hypothyroidism (Duntas & Maillis, 2013; Hage & Azar, 2012) or Cushing's syndrome (Arnaldi et al., 2003; Pivonello et al., 2016; Sonino, Fava, Raffi, Boscaro, & Fallo, 1998)]. However, beyond clinical consensus, there is little empirical guidance on the relative costs and yield of these potential diagnostic tests and procedures. Relevant issues include whether assessment algorithms are specified by symptomatic presentation, treatment history, or sociodemographic characteristics. Similarly, evidence is currently incomplete regarding the potential of pharmacogenetic/genomic testing to identify or subgroup DTD (Vittengl, Clark, Thase, & Jarrett, 2019; Zeier et al., 2018). How these issues are resolved will impact the inclusion and exclusion criteria used by intervention researchers to define DTD.

### Define the boundaries and develop a taxonomy for DTD

The current clinically based characterization of DTD (Gaynes et al., 2018, 2020) lacks sufficient specificity to define intervention study subpopulations. The challenges to defining DTD subpopulations are complex: persons with DTD are heterogeneous in treatment history and responsiveness, sensitivity to treatments, prognosis, and pathobiology (McAllister-Williams et al., 2020; Rush et al., 2019). Clinical research with DTD would benefit from an evidence-based taxonomy that can identify more homogeneous subgroups. Such a taxonomy would make intervention research more cost-efficient and potentially improve our ability to match specific interventions with DTD subgroups. These empirically defined subgroups would also assist mechanistic researchers in elucidating the various pathobiological pathways that likely underlie different types of DTD.

To illustrate the need for a DTD taxonomy, consider the STAR*D findings that acute response and remission rates decreased with increasing numbers of failed treatment trials (e.g. 48.6%, 28.5%, 16.8%, 16.3% for the first four acute phase treatment attempts, respectively). In addition, when remission was achieved, relapse/recurrence rates during follow-up increased progressively with more previously failed acute phase treatments (Rush et al., 2006b; Sackeim, 2016). It is unknown whether these two findings reflect unitary or distinct neurobiological effects linked to antidepressant treatment resistance. Indeed, within DTD, some individuals show minimal treatment responsivity despite repeated interventions, while others experience substantial short-term benefit, but do not stay well. Are these etiologically distinct groups that may require different treatment strategies? A similar concern can be raised when considering individuals with a chronic course with few or no intervening periods of wellness compared to individuals with recurrent, relapsing depression. A course of illness seems to differentially relate to acute and longer-term treatment outcomes in depression (Rush et al., 2012).

Our current working taxonomy for DTD is based on the designation, TRD, which itself is highly variable in operationalization (Berlim & Turecki, 2007a; Gaynes et al., 2018, 2020). TRD is typically ascribed after two unsuccessful, but well-delivered, acute phase treatments, a threshold that is supported by the marked decrease in sustained remission rates in STAR*D after the first two treatment trials (Conway et al., 2017; Rush et al., 2006b).

This empirical model of TRD, however, is not an adequate taxonomy for DTD. Any classification/treatment algorithm of DTD must consider multiple DTD variations. For example, DTD patients who are hypersensitive to medication side effects and cannot tolerate two medication trials may be difficult-to-treat, but are not ‘treatment resistant’. DTD patients who receive interventions and show marked transitory benefit which is not sustained are not considered ‘treatment resistant’ (Gaynes et al., 2018; Sackeim et al., 2019), but clearly are difficult to treat. In addition, the nature of the two failed treatments is not specified (e.g. two selective serotonin reuptake inhibitors (SSRIs) v. one SSRI followed by transcranial magnetic stimulation) (Fava, 2003b; Fekadu et al., 2009), and it is unlikely that all treatments are equivalent in their prognostic implications for insufficient or short-lived benefit.

This dilemma could be addressed, in part, by staging the ‘level of resistance’ (analogous to cancer staging) (Conway et al., 2017; Thase & Rush, 1995), based either on a minimum number of *specific treatment types* (e.g. monoamine reuptake inhibitors; brain stimulation treatments, depression-targeted psychotherapies) or *specific treatment sequences* [e.g. cognitive behavioral therapy to SSRI to atypical antipsychotic augmentation to monoamine oxidase inhibitor to electroconvulsive therapy (ECT)]. Even with this approach, the introduction of a new treatment will change sample definitions. Further, such staging approaches focus only on treatment history rather than the broader clinical context and course.

### Characterizing DTD: Clinical features, perpetuating factors, and temporal evolution

**Fig. 1.**
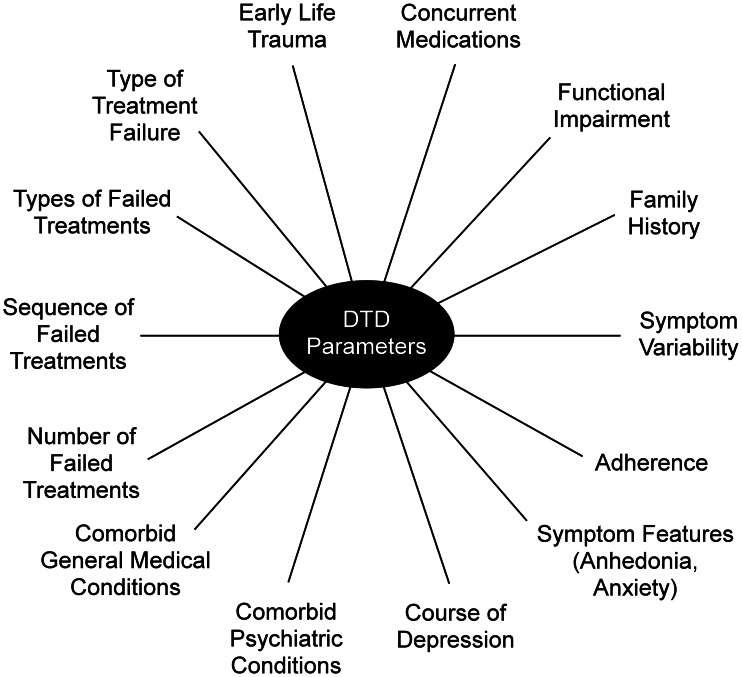
Potential parameters to define DTD or to characterize subgroups.

A more fruitful approach may be a multidimensional characterization of DTD based on its associated clinical presentations, clinical course, biomedical, prognostic, neuropsychological, treatment response, and other features. The degree and duration of functional impairment, history of symptomatic improvement and relapse, and presenting symptoms may be as consequential in identifying and characterizing DTD as the number and type of unsuccessful treatment attempts. These parameters would form the basis for an evidence-based DTD taxonomy that would not necessarily change as new treatments or management tools arrive. These features would define the overall DTD population, and inform the identification of subgroups or spectrums (with or without the addition of biomarkers or pharmaco-dissection). This effort could begin by evaluating whether specific features distinguish DTD from non-DTD patient groups (e.g. concurrent general medical problems; types and severity of anxiety symptoms; types, severity and chronicity of environmental stressors; substance use/abuse disorder; history of childhood trauma/abuse; etc.) (McAllister-Williams et al., 2020; Rush et al., 2019) (Fig. 1).

DTD raises additional challenges to taxonomy development. It is likely that psychosocial determinants of health are perpetuating factors, and these must be addressed to achieve better outcomes, including chronic occupational, marital, economic, or health stressors; co-morbid substance misuse; sedentary lifestyle; obesity; etc. In addition, predisposing developmental factors, such as childhood trauma, may continue their impact on DTD by impairing resilience and problem-solving. These social determinants may alter both the risk of developing and the likelihood of maintaining/recovering from DTD (Holzel, Harter, Reese, & Kriston, 2011; Negele, Kaufhold, Kallenbach, & Leuzinger-Bohleber, 2015; Verhoeven et al., 2020).

Although a single episode of depression that is responsive to treatment is unlikely to ‘scar’ personality (Shea et al., 1996), years of unremitting depression – with or without adequate treatment – may alter the clinical course and have taxonomic implications for DTD. Persons with DTD may develop behavioral and thought patterns that exacerbate their negative self-valuation and pessimism. In essence, the depression builds on itself, intensifying self-defeating thought patterns and worsening the condition. Such ‘secondary’ changes in morale, self-efficacy and perceived resilience or grit may provide therapeutic opportunities (Thase & Howland, 1994; Young, 2018), as in helping patients with DTD to identify psychological ‘negative feedback loops’ and trigger the use of specific psychotherapeutic strategies to mitigate their impact (Eisendrath et al., 2015; Lynch et al., 2020).

The years of hopelessness associated with DTD may alter its course and have taxonomic implications. Persons with DTD may develop behavioral and thought patterns that exacerbate their negative self-valuation and pessimism. In essence, the depression builds on itself, intensifying self-defeating thought patterns and worsening the condition. This secondary demoralization may provide therapeutic opportunities. For example, identifying psychological ‘negative feedback loops’ could trigger the use of specific psychotherapeutic interventions to mitigate their impact.

The consideration of a DTD taxonomy raises concern about whether the psychology and biology of DTD evolve over time, as appears true in multiple medical conditions (e.g. congestive heart failure, atrial fibrillation, cancers). For many DTD patients, treatments effective earlier in their illness subsequently lose benefit, suggesting a developmental change in key neurobiological substrates (Katz, 2011). This observation also leads to a related and worrisome consideration: the possibility that exposure to ineffective or partially effective treatment induces neurobiological change such that treatment responsiveness diminishes and the depression becomes more difficult to treat (Andrews & Amsterdam, 2020; Andrews, Kornstein, Halberstadt, Gardner, & Neale, 2011; Fava, 2003a; Sackeim, 2016). In other words, treatment resistance could beget more profound treatment resistance and greater chronicity. This possibility illustrates one of the many complex research challenges posed by DTD in developing a taxonomy.

### Assessment of antidepressant treatment history

Knowledge about the nature and results of prior depression treatments is crucial to informing a taxonomy. This information (e.g. dose, duration, adherence, outcome) will be key in distinguishing among patients who do not receive ‘adequate’ therapeutic trials due to intolerance, those who show only minimal acute benefit despite ‘adequate’ treatment, and those who have greater acute responsivity but cannot sustain the benefit. At a practical level, such knowledge could also inform investigators as to which patients have already benefited or not from the intervention under study. In theory, a national electronic health record (EHR) would be optimal in providing universal and uniform data collection (Fife et al., 2017; Gronemann, Jorgensen, Nordentoft, Andersen, & Osler, 2020), but the USA is decades from that possibility. An alternative approach of melding different EHRs to create a continuous treatment narrative is feasible in principle, but a major challenge and expense.

To overcome these challenges, the field has developed several tools to retrospectively gather and evaluate treatment history, including the Antidepressant Treatment History Form (Sackeim, 2001; Sackeim et al., 2019), Maudsley Staging Method (Fekadu et al., 2009), and Massachusetts General Hospital-Antidepressant Treatment Questionnaire (Chandler, Iosifescu, Pollack, Targum, & Fava, 2010), among others (Gaynes et al., 2018). However, obtaining the requisite information is labor-intensive given the fractured nature of our health care systems, and the fact that past providers, pharmacies, medical facilities, patients, families, and caregivers may have differing and useful information that must be integrated. The rigor in establishing prior history, and thus the quality of the information obtained, likely differs among studies, which reduces consistency in findings. Furthermore, these tools have not been compared, and extensive validation studies have not been undertaken. Thus, while the assessment of treatment resistance is an integral part of DTD characterization and has shown predictive power regarding responsivity to subsequent treatment (Heijnen et al., 2010; Lisanby et al., 2009; Rush et al., 2006b) and relapse potential (Prudic et al., 2013; Sackeim et al., 1990), the assessment tools require further development.

## Selecting, acquiring, and interpreting outcomes in DTD

### Selecting among DTD outcomes

**Fig. 2.**
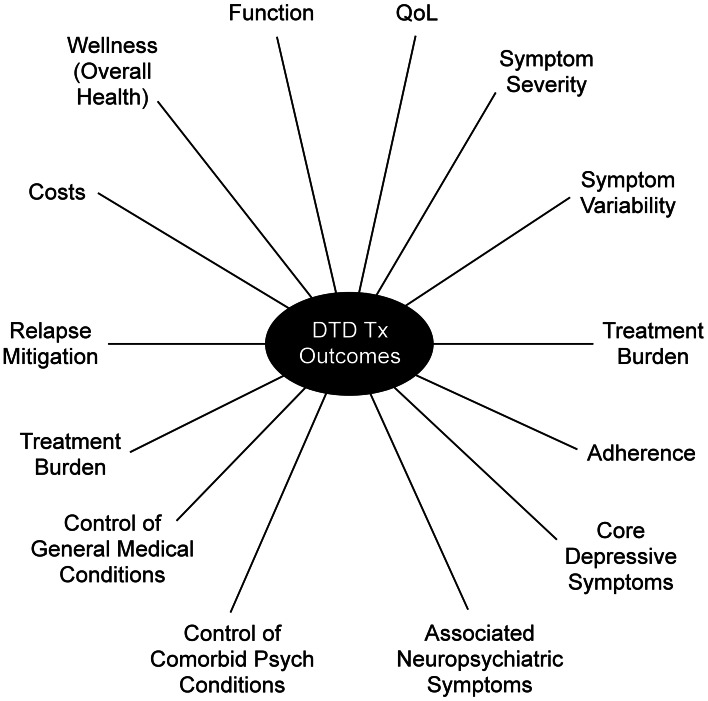
Clinically important outcomes for DTD intervention research. Psych = Psychiatric; Tx = Treatment.

Until now, the primary outcome metric for evaluating MDE interventions has been depressive symptom severity, assessed over 6–12 week acute trials, and quantified either by a change in scores on a clinician-rated scale or by the proportion of participants with a clinically meaningful benefit specified categorically (e.g. remission, response, partial response) compared to control conditions. However, depressive symptoms may not necessarily be the most critical outcome. Some modest, but valuable, degree of symptom control has often already been achieved, and further meaningful symptomatic reduction is not expected, given the history of responsivity to prior treatments. Aside from symptom control, DTD treatments/interventions may aim to improve other factors, such as managing concurrent psychiatric and general medical conditions, minimizing treatment burden, enhancing daily function, QoL, or overall mental and physical wellness, mitigating symptomatic worsening, or otherwise reducing mood instability (Fournier, DeRubeis, Amsterdam, Shelton, & Hollon, 2015; McAllister-Williams et al., 2020; Rush & Thase, 2018; Rush et al., 2019) (Fig. 2). From the care system-resource management perspective, cost efficiency is also an important DTD outcome, given its chronicity (Ross, Zivin, & Maixner, 2018).

To illustrate the potential importance of these holistic outcomes, patients who receive ECT are particularly characterized by their level of baseline impairment in social and vocational function and QoL. These deficits are typically as or more critical than symptom severity in leading to ECT referral, and typically resolve fully only with sustained remission following ECT (McCall, Prudic, Olfson, & Sackeim, 2006; McCall et al., 2013, 2017). Nevertheless, ECT trials have focused exclusively on the level of depressive symptom reduction as the primary outcome.

### Choice of primary and secondary outcomes

A wide range of outcomes (with or without symptoms) can be targets for DTD interventions (Fig. 2). Researchers face the challenge of prioritizing amongst them. A single intervention may be aimed at one or more targets (e.g. symptom control and daily function). However, these various potential therapeutic outcomes may manifest at different times (e.g. symptom control may precede functional improvement by weeks or months) (Hofmann, Curtiss, Carpenter, & Kind, 2017; Paykel, 2002). There may also be trade-offs between achieving one goal (e.g. minimizing treatment burden) and optimizing function (Fournier et al., 2015).

Selecting a primary outcome would be far simpler had we an analog in DTD of the hemoglobin (Hgb) A1C measure in diabetes. This measure reflects the average glucose level in the bloodstream over the prior 2–3 months (thereby taking into account complex effects of multiple determinants). Hgb A1C is a strong indicator of disease process/control, with established benchmarks for normal, moderately severe, and severe dysregulation. It informs management decisions, and assessment of disease outcomes and complications (Sherwani, Khan, Ekhzaimy, Masood, & Sakharkar, 2016). Unfortunately, DTD is likely more heterogeneous with respect to etiology and pathophysiological processes, treatment responsiveness, and other factors, so it is unlikely that a single measure like Hgb A1C will emerge. Such a measure may be achievable within a specific DTD subgroup.

Symptom control in DTD refers to control of core criterion depressive or manic symptoms, as well as associated symptoms which can impact QoL, day-to-day functioning, and relapse risk. Such symptoms may include insomnia, pain, anxiety, irritability, cognitive impairment, and substance misuse. Which DTD outcome measures are chosen depends on the question(s) being addressed and the specific DTD subgroup under study. A reduction in symptomatic variability (waxing and waning) may be especially important in the management of the difficult-to-treat bipolar disorder, while addressing treatment burden and adherence may be key outcomes in another subgroup. We recognize that bipolar depressions are also often difficult-to-treat and the research and clinical challenges posed by bipolar DTD deserve separate in-depth discussion.

To enhance day-to-day function and QoL in DTD, optimizing control of medical and psychiatric co-morbidities may also be prioritized. This imposing number of potential primary and secondary outcomes in DTD presents challenges in selecting assessment domains and specific measures, and in determining assessment frequency. Therefore, it is essential that studies are based on specific hypotheses that determine the outcome measures chosen.

### Are multi-dimensional or composite outcomes needed for DTD?

The multifaceted outcomes in DTD raise the possibility of approximating a ‘Hgb A1C-like’ outcome metric by forming a composite or multidimensional outcome measure from the assessment of several diverse outcomes. Composite outcomes typically combine various aspects of a particular single construct, such as speed and extent of symptom change, or likelihood and persistence of such change. Multidimensional outcomes assess diverse domains that cannot be easily reflected in a single construct, such as the benefits and costs of an intervention (Schwartz & Patrick, 2014).

**Fig. 3.**
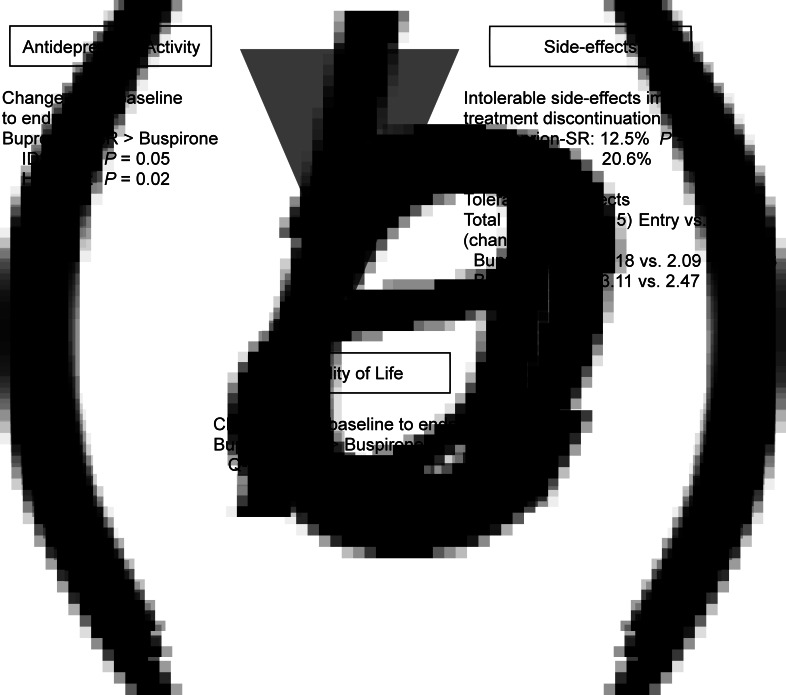
Application of the pharmaco-psychometric triangle. *Note*: Figure recreated from Bech et al. (2012). HAM-D6 = Hamilton Rating Scale for Depression 6-item subscale; IDS-C6 = Inventory of Depressive Symptomatology 6-item subscale – Clinician-rated; PRISE = Pragmatic-explanatory continuum indicator summary; Q-LES-Q = Quality of Life Enjoyment and Satisfaction Questionnaire; SR = Sustained release.

Figure 3 illustrates an attempt at evaluating outcomes using a multidimensional approach (Bech, 2009; Bech, Fava, Trivedi, Wisniewski, & Rush, 2012). Bech et al. (2012) applied the ‘pharmaco-psychometric triangle’ which includes three dimensions that, when presented separately, enable clinicians and patients to see the trade-off between benefits and side effect burden (Bech, 2009). This approach meaningfully differentiated between buspirone and bupropion as adjunctive agents in depressed outpatients – a difference that was not observed when comparing effects on depressive symptoms alone (Trivedi et al., 2006).

Composite or combinatorial outcomes may have particular prognostic value in depression, whether or not specific to DTD. The utility of combinatorial measures was illustrated by Cohen, Greenberg, and IsHak (2013). In a retrospective analysis of the STAR*D database, they found that three measures (symptom severity, function, and QoL) combined into a ‘burden of illness scale’ was better at predicting time to relapse following successful acute-phase antidepressant treatment than any element alone.

Regardless of whether standard scales, new composite, or new multidimensional measures are adopted as outcome metrics for DTD intervention studies, we must be able to translate changes in such scores into clinically useful categories that facilitate clinical decision-making and are meaningful to patients, care systems, and clinicians. This aim is analogous to the outcome categories of partial response, response, and remission, based on symptom change used in traditional acute antidepressant trials. For DTD patients, for whom various interventions with diverse aims (e.g. function, QoL, treatment burden, etc.) may be attempted, stakeholders will want to know what incremental changes are likely to be achieved in which groups of patients. Such a multidimensional/combinatorial model for DTD will require consensus as to what represents positive clinical outcomes.

### How often and when should outcomes be obtained?

Typically, longitudinal outcome data address three aspects of change: (1) average change in the population; (2) individual differences in the degree of change; and (3) individual within-person change over different temporal intervals (Hofer, Thurvaldsson, & Piccinin, 2012). Therefore, the primary and secondary aims of a study must inform decisions regarding the frequency of obtaining outcomes of interest.

Some treatments are expected to differ radically in time to the onset of clinical effects. For example, symptomatic change with (es)ketamine may be observed within hours of the first infusion and, unless treatment is continued at regular intervals, usually wanes over the next 5–7 days (Zarate & Niciu, 2015). Other interventions, such as Vagus Nerve Stimulation, require substantially longer time frames (months to years) to fully manifest therapeutic benefit (Aaronson et al., 2017; Berry et al., 2013). Therefore, the frequency of repeated assessments should take into account variation in the outcome measure within and across individuals, the treatment being studied (Hofer et al., 2012), and the aim of treatment (e.g. acute symptom control or longer-term prophylaxis).

Different outcomes may need to be collected at different times because the time course for achieving these potentially important, but diverse, objectives is variable. For example, depressive symptom improvement often occurs before the full realization of improvement in function/QoL (McKnight & Kashdan, 2009). Given the temporal variability in symptom expression and the high rate of relapse following initial improvement, the durability of benefit is a key consideration in evaluating the therapeutic effects in DTD (Jelovac, Kolshus, & McLoughlin, 2013; Rush et al., 2006b; Sackeim et al., 2007) and considering the relative merits of alternative interventions (Cuijpers et al., 2013). Thus, the choices of outcomes, and when and how often they are measured, should be paramount when determining the duration of DTD treatment trials.

In addition, there can be substantial day-to-day variation in symptoms, function, and other outcome domains in DTD, either spontaneously or in reaction to environmental events. Furthermore, relatively small degrees of change consistently maintained may be salutatory and even the main goal of treatment. However, detection of such limited degrees of change is highly contingent on the reliability of the baseline measures against which all subsequent assessments are compared. It is critical to allow adequate time (perhaps weeks) with potentially multiple averaged measures to establish a true baseline in DTD. Thus, the frequency of outcome assessments and the duration of the baseline period may be especially critical considerations when designing DTD intervention studies.

### Which sources provide the most valid outcomes for DTD intervention research?

In typical acute-phase antidepressant trials with pharmacotherapy, psychotherapy, or ECT, there is a relatively moderate correlation between baseline self-report and clinician-ratings of symptom severity, often on the order of only 25% shared variance (Sayer et al., 1993; Uher et al., 2012). Of note, effect sizes for therapeutic interventions in MDEs are typically larger for clinician ratings than self-reports (Sayer et al., 1993). Such discrepancies may be both larger and more likely in DTD for which self-appraisal distortions or thinking biases caused by chronic illness or awareness of chronic shortcomings may further exaggerate negative self-assessment and impaired motivation. It is the common clinical experience for chronically depressed patients to be slower to recognize symptomatic improvement than clinicians who observe them at regular intervals. This suggests that the validity of self-report and the use of both self-reports and clinician ratings as combined outcomes in DTD deserve study. Older depressed persons show a consistent tendency to under self-report symptom severity relative to observer measures (Fiske, Wetherell, & Gatz, 2009; Gallo, Anthony, & Muthén, 1994). It is unknown whether this aging effect is maintained, reversed, or accelerated in DTD, but given that DTD frequently persists well into old age, this factor must be considered. It would also be valuable to explore the utility of combining self- and observer-rated scales into a composite measure.

### Early identification of mediators, moderators and predictors is essential

Due to major evidence gaps, clinicians treating individuals with DTD face several decision-making challenges, including (1) choosing among alternative treatments for a specific patient (identifying prescriptive predictors based on moderator analyses); (2) estimating the likelihood of benefit of a single treatment (based on baseline features, i.e. prognostic predictors); and (3) identifying mediators of treatment outcome [i.e. processes deemed to be essential to achieving the benefit (Kazdin, 2007)].

For example, consider a treatment aimed at enhancing psychological abilities to promote resilience-enhancing skills in everyday life, which in turn is expected to improve daily function or QoL. The measurement of resilience (the hypothetical mediator) is essential to determine whether the treatment outcomes achieved actually depend on enhanced resilience in those who benefit.

The identification of mediators, moderators, or prognostic predictors is especially important in DTD due to its etiological and treatment response heterogeneity. Any intervention will likely be useful for only a subset of DTD patients. Some outcomes may entail different mediators, moderators or prognostic predictors than others. From a cost-efficiency perspective, it is less expensive to select candidate measures as potential predictors and mediators early in the conduct of trials, whether observational or randomized, given the overall cost of conducting the trial (Trivedi et al., 2016; Uher et al., 2009). The identification of any mediator, moderator, or prognostic predictor could contribute to the taxonomy and to targeting the treatment to those most likely to benefit.

### How should outcomes be collected?

To contain costs and gain granularity, we may need to consider different ways to acquire outcomes, especially to assess within-person change. This granularity will be critical when identifying mediators of change that gain traction at different time periods across different patients. For example, if reduced symptomatic variability is a mediator for improved QoL/daily function, this relationship may appear earlier in the course of some treatments than others. In addition, within a single treatment, some may show the response and thus reveal the mediator earlier than those who respond later. This variation across patients demands a level of granularity for outcome acquisition that can prove cost-prohibitive unless cleverly designed. Hypothetically, remote sampling via smartphones and passive collection of behavioral and physiological data could provide more precise and frequent outcome sampling. Indeed, intervention studies of DTD in the real world could be improved by delinking outcome assessment from treatment visits. Such visits are often scheduled based on how well the patient appears and other clinical considerations that, ideally, should be independent of the timing of outcome measurement. With the rise in telepsychiatry and data acquisition facilitated by natural language processing (NLP) and other forms of artificial intelligence (AI), remote real-world sampling of outcomes (e.g. symptoms, function, or QoL) has become cost-effective and potentially may provide more valid outcomes.

## Challenges in intervention trial design

This section discusses three trial design challenges presented by DTD: sample sourcing, trial execution, and intervention study designs that preserve causal inference.

### Sample sourcing and eligibility criteria

DTD and TRD are heterogeneous in clinical presentation, course of illness, biology, treatment responsivity, and other factors. Recruiting DTD intervention research participants from representative real-world treatment settings (as opposed to research clinics) would help ensure a representative sample which enhances the generalizability of findings and may reduce costs of recruitment, treatment delivery, and trial management costs. Large care systems, whether governmental (e.g. VA) or private commercial systems, can lower costs by providing much of the information on the prior treatment of mental health and general medical conditions.

Access to large numbers of representative participants helps to address the heterogeneity issue. Large numbers enable the identification of subgroups within the DTD patient population, especially those responsive or unresponsive to a particular intervention. This enables post-hoc secondary analyses to generate hypotheses for subsequent studies. Larger patient populations also increase the certainty of findings which, when obtained in real-world settings, enable rapid real-world implementation.

However, due to the heterogeneity of DTD and our current lack of taxonomy, there are risks in being too inclusive. Inclusion criteria must ensure that people with the targeted problem are included with few others, while the exclusion criteria might best be minimal to enhance generalizability. For example, a treatment targeting anhedonia in DTD might include depressed patients with sufficient baseline anhedonia to show the intended effect. In addition to the heterogeneity of DTD, potential efficacy-confounding aspects of DTD (e.g. the presence of a personality disorder, a background of substance misuse) are often not easily identifiable by standard review of diagnoses/medication exposure in large EHR databases. This is particularly challenging as concomitant personality disorder and substance addiction significantly impact depression outcomes (Davis et al., 2006; Mulder, 2002). For the particular trial, the judicious assessment of factors that are suspected of affecting the outcome could be acquired on a full or subsample basis for secondary analyses.

### Trial execution

To acquire large samples of representative DTD patients, point-of-care trials at multiple sites would be preferred with an emphasis on larger numbers of participants and, to contain costs, modest numbers of process and outcome measures (Fiore et al., 2011; Shih, Turakhia, & Lai, 2015). Simple self-reported outcomes, perhaps collected via smartphones and/or at clinic visits (e.g. symptom burden, treatment burden/side effects, and function/QoL), can be both reliable and cost-efficient. Conveniently and frequently acquired global ratings may be as informative as measures derived from longer questionnaires, especially self-ratings on items or item response patterns that yield clinically meaningful outcome differences (Turkoz et al., 2021).

One strategy to identify the target sample for new interventions in DTD is the use of patient registries, in which initial testing of an intervention in an open trial identifies the subgroups for which the intervention is more or less effective (Aaronson et al., 2017; Sackeim et al., 2020). These registries would obtain patient-reported outcomes on a wide swath of DTD patients. Such information would form the basis for an evidence-based selection of a limited number of inclusion and exclusion criteria for use in subsequent randomized trials. This sort of registry would also identify individuals for whom the intervention causes untoward effects or no meaningful benefit, thereby enriching the study sample and reducing costs in the randomized controlled trial. In addition, preferred dosing and the expected trajectory of benefit would support a more time and cost-efficient randomized controlled trial design.

### Study designs to optimize both generalizability and causal inference (hybrid trials)

**Fig. 4.**
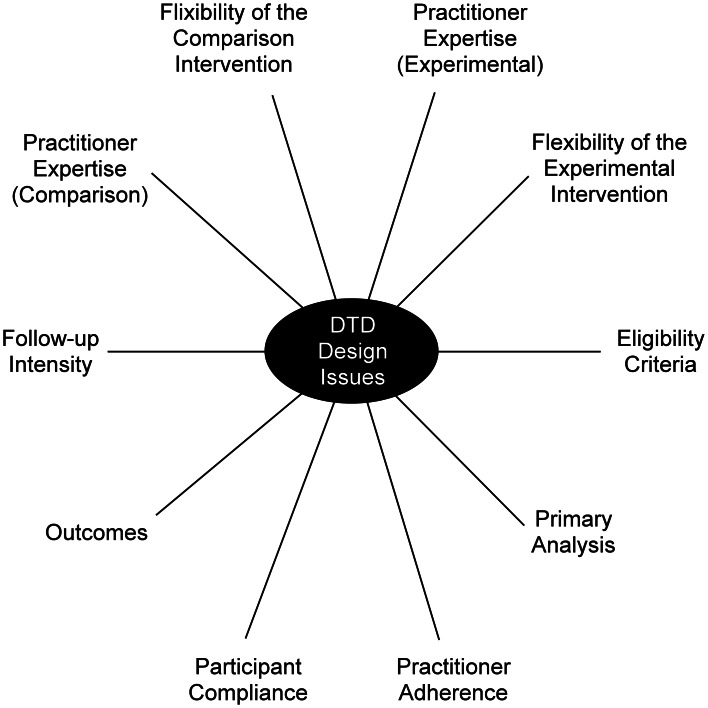
Pragmatic-explanatory continuum indicator summary (PRECIS) wheel (Thorpe et al., 2009).

A study of representative DTD patients treated in real-world settings raises a range of trial design considerations. Thorpe et al. (2009) identified 10 treatment trial design parameters that likely affect outcomes, and which vary considerably between highly controlled efficacy (explanatory) studies and effectiveness (pragmatic) studies. They include practitioner expertize in the interventions, eligibility criteria, follow-up frequency, outcomes, patient compliance, practitioner adherence, and flexibility of the experimental and comparison interventions (Fig. 4). These parameters are worth considering since variable delivery of the intervention(s) in relation to these parameters has untoward consequences: (1) a very large sample may be needed to detect treatment effects; (2) ability to detect a signal (intervention difference) is reduced; and (3) if a difference is found between the interventions, the cause may be due to one or more of these parameters differentiating the two groups rather than differences in intrinsic therapeutic properties. Controlling these parameters in trial design can increase the certainty of inferring that the between-intervention differences (or non-differences) are due to the interventions and not an artifact of variability in delivery or selection bias.

This strategy enables causal attributions to be made when between-group differences are found in specified comparisons. For example, STAR*D engaged representative patients and practitioners while controlling the delivery of treatment using a measurement-based care guidance approach (Rush et al., 2004). This helped ensure that when treatment was unsuccessful, it was the treatment that failed and not its delivery. To make causal inferences from real-world studies, investigators should consider which parameters should be controlled, which will be assessed, and consequently, what kinds of causal inference can be made (Fig. 4).

## Conclusions

In recent years, our expectations about the clinical utility of antidepressant medications and psychotherapies in depressive and related mood disorders have been lowered, particularly for individuals who have a history of nonresponse to several standard forms of therapy (Cuijpers, Karyotaki, Reijnders, & Ebert, 2019; Penn & Tracy, 2012). This expectational shift became apparent as intervention research moved to real-world patients with broader inclusion and fewer exclusion criteria, and as treatment delivery became less controlled (Bauer et al., 2009; Rush et al., 2004; Uher et al., 2012). It is now recognized that only about one-third of those who receive an initial course of antidepressant pharmacotherapy will experience a sustained remission. Furthermore, previous treatment failure decreases the likelihood of achieving acute remission at the end of subsequent short-term medication trials, while also increasing the likelihood of relapse if remission is achieved (Conway et al., 2017; Rush et al., 2006b; Sackeim, 2016).

Despite the profound personal, familial, and societal costs of DTD, patients with DTD are often excluded from studies of therapeutic interventions and neurobiology. Even trials that specifically address therapeutics in TRD often cap the number of failed prior treatment trials or the duration of the current episode precisely because greater treatment resistance or chronicity is expected to negatively impact therapeutic outcomes, limiting detection of a therapeutic signal (O'Reardon et al., 2007; Rush et al., 2005). DTD patients are also excluded from research because their high level of medical and psychiatric co-morbidities, suicidality, or functional impairment may present safety concerns, complicate identification of therapeutic signals, or impose practical impediments to research participation.

Other branches of medicine have identified subgroups that do not benefit sufficiently from standard therapeutics and have developed methods to specifically study these populations and identify novel treatments or interventions that mitigate aspects of the clinical presentation. For example, consensus guidelines in epilepsy recommend consideration of surgical or neuromodulatory interventions following two unsuccessful trials of anticonvulsant medications (Brodie et al., 2012; Callaghan et al., 2007; Jette et al., 2014; Kwan & Brodie, 2010). The European League Against Rheumatism defines difficult-to-treat rheumatoid arthritis (D2T RA) as persistent signs and/or symptoms despite a trial of two or more biologic or targeted synthetic disease-modifying anti-rheumatic drugs with different mechanisms of action, signs suggestive of active/progressive disease, and disease management viewed as problematic by the clinician and/or patient. Multiple differences have been identified in clinical presentation, treatment burden, and co-morbidities between D2T RA and comparison patients, and empirically defined subgroups within D2T RA have been proposed (Roodenrijs et al., 2021). In contrast, we lack information on when in the course of multiple treatment trials DTD should be declared, with altered expectations regarding prognosis and the types of interventions considered. Indeed, since DTD has not been an object of study, we lack fundamental information on the nature and size of this population, demographic and clinical characteristics, optimal management, and long-term outcomes.

In conclusion, the traditional research methods used to develop and test therapeutic interventions in treatment-responsive or treatment-naïve mood disorder populations are often inapplicable in DTD. Indeed, our understanding of the natural history of mood disorders, the phases of illness, and the phases of treatment (e.g. continuation or maintenance regimens) may not readily apply to DTD (Frank et al., 1991; Rush et al., 2006a). Nonetheless, many individuals do not achieve sustained remission despite multiple well-delivered treatments. These individuals consume a disproportionate share of health resources, while experiencing a disproportionate degree of functional impairment and prolonged suffering. Intervention research is sorely needed and should be particularly useful if it takes into account the clinical research challenges posed by DTD.
